# Modality preferences for health behaviour interventions for post-treatment cancer survivors: a theoretical investigation

**DOI:** 10.1007/s00520-023-07607-8

**Published:** 2023-02-02

**Authors:** Morgan Leske, Bogda Koczwara, Julia Morris, Lisa Beatty

**Affiliations:** 1 College of Education, Psychology, and Social Work, Flinders University, Adelaide, SA Australia; 2 College of Medicine and Public Health, Flinders University, Adelaide, SA Australia; 3 Department of Medical Oncology, Southern Adelaide Local Health Network, Adelaide, SA Australia; 4 Cancer Council SA, Eastwood, SA Australia

**Keywords:** Lifestyle intervention, Cancer survivors, Digital health, Telephone, Preferences

## Abstract

**Purposes:**

User preferences for how programs are delivered are an important consideration when developing healthy living interventions. The aim of this study was to investigate (a) if cancer survivors prefer telephone or internet delivery for a healthy living intervention and (b) what factors were associated with delivery preference.

**Methods:**

Australian cancer survivors (18 + years) were invited to complete an online or hardcopy cross-sectional survey measuring social and clinical demographic factors and validated measures of self-efficacy, health literacy, and social support.

**Results:**

Of the 168 respondents, the majority were female (*n* = 147, 92%) and breast cancer survivors (*n* = 122, 80%) and preferred internet delivery (*n* = 109, 65%). Participants who preferred internet delivery had a longer time since diagnosis (*M* = 9.85 years, *SD* = 8.20) compared to those who preferred telephone (*M* = 6.80 years, *SD* = 5.54), *p* = .03. However, logistic regression analyses demonstrated that no other variables (age, gender, socio-economic status, BMI, education, self-efficacy, health literacy, nor social support) had a direct association on delivery preference.

**Conclusions:**

Cancer survivors appear to prefer internet delivery to telephone, particularly for those further along the survivorship trajectory. Future intervention development should therefore consider the internet modality for delivering accessible health interventions and offer the program to long-term cancer survivors. Whether these findings are replicable in the current post-pandemic phase is an important avenue for future research.

**Supplementary Information:**

The online version contains supplementary material available at 10.1007/s00520-023-07607-8.

## Introduction

Regular physical activity, maintenance of healthy weight, and adequate nutrition can improve quality of life after cancer treatment, by supporting well-being and reducing the risk of treatment-related side effects, cancer recurrence, and comorbidities [1, 2]. Despite these benefits, many Australian cancer survivors are not meeting the healthy lifestyle recommendations outlined by national cancer support organisations [3, 4]. This may be due to lack of awareness, lack of motivation, or lack of guidance and support from healthcare professionals [5]. While face-to-face interventions have demonstrated efficacy for increasing health behaviours, these interventions are costly and are not available to all survivors, especially those in geographically isolated areas, as they encounter mobility constraints to accessing health services and have fewer available services where they reside [5]. Furthermore, the recent COVID-19 pandemic has demonstrated that face-to-face delivery can be subject to disruption [6]. Consequently, there is a growing interest in more accessible intervention delivery modalities, namely telephone and online platforms, with emerging evidence supporting their efficacy and acceptability [7].

Telephone-delivered interventions involve participants engaging with a coach or healthcare professional via phone calls in which they receive health behaviour guidance and establish health-related goals [8]. While telephone interventions have demonstrated efficacy in supporting clinically significant improvements in physical activity, dietary behaviours, physical quality of life, and cancer-related symptoms [9], this modality is susceptible to sustainability barriers. Comparatively, internet-delivered interventions can integrate dynamic elements that users can engage with at any time to support the establishment and achievement of health-related goals [10]. While internet delivery may enhance program sustainability by requiring minimal financing and resources following its development, whether this meets the preferences of cancer survivors has been surprisingly under-explored.

Understanding end-user preferences for mode of intervention delivery—that is a preference for telephone vs internet—is an important feasibility consideration for developing interventions. By understanding participant preferences, the design and implementation of an intervention can be tailored to suit the profile of users. Such tailoring may increase uptake, adherence, and satisfaction towards the intervention [11]. Currently, there is a dearth of research focused on predictors for intervention modality preference for health behaviour interventions. Most studies exploring modality preferences (a) either have utilised qualitative methodology, finding that cancer survivors desire an accessible intervention modality for privacy and convenience [12, 13], or (b) have briefly measured modality preference alongside content preferences [14, 15]. Of the research that has explored correlates of delivery modality preferences, factors explored thus far include demographics (i.e., age, gender, income, education, and access to computer) and clinical (i.e., body mass index (BMI) and time since diagnosis) and current health behaviours (i.e., physical activity and fruit and vegetable consumption). Collectively, these studies have demonstrated that socioeconomic status (SES) and education are positively associated with internet delivery preference, whereas older age and higher BMI are positively associated with telephone delivery preference [16–19]. Research on the association between gender and modality preference has produced mixed findings [19, 20].

Although this research offers a starting point, it provides a limited understanding of *how* these demographic factors might impact preference. A recent systematic review on patient preferences and decisions in healthcare settings suggests that preferences might be influenced by personal, social, and cognitive factors [21]. Social cognitive theory (SCT) has been frequently utilised in the development of health interventions and offers a useful framework for considering how demographic factors influence delivery modality preference. The theory outlines a core set of determinants that influence how health knowledge is translated into effective health practice, including knowledge of health benefits and risks, perceived self-efficacy, outcome expectations, health goals, and perceived facilitators and barriers (e.g., social support) [22]. Of these factors, the determinants of self-efficacy, health literacy, and social support have demonstrated preliminary empirical support for their relevance to modality preferences. More specifically, high levels of self-efficacy, health literacy, and social support have been associated with using additional resources, such as the internet, to seek health information [23].

Thus, the objectives of the present study were to build on these findings and explore (a) whether the SCT factors of self-efficacy, health literacy, and social support are associated with preference for internet over telephone delivery of a healthy living intervention and (b) whether these factors mediate the relationship between established sociodemographic factors (SES, education, age, and BMI) and modality preferences.

## Methods

### Participants

Participants were adults (aged ≥ 18 years) living in Australia with a personal history of cancer and had completed active cancer treatments (e.g., surgery, chemotherapy, and radiotherapy), except for individuals on hormone treatment, who were still eligible. The study was advertised through paid and unpaid “organic” posts on Facebook, and a flyer distributed at local cancer support group meetings and accommodation services provided by Cancer Council SA, and at fundraising events hosted by Cancer Council SA. Further, information about the survey was disseminated in Breast Cancer Network Australia’s Review and Survey group.

As a new measure for modality preference was developed for the current study, an effect size could not be derived from previous research to conduct a power analysis. Instead, the rule of thumb of 10 events per variable was used to guide our targeted sample size, resulting in a target of 80 participants per modality preference group [24]. Ethics approval was obtained via Cancer Council Victoria’s Human Research Ethics Committee (#IER1904).

### Design and materials

A cross-sectional survey design was used, encompassing demographic questions and a battery of psychometrically validated and internally reliable measures. Participants completed the survey either online or via a hardcopy disseminated in person.

#### Sociodemographic information and clinical history

Sociodemographic items included age, gender, ethnicity (i.e., country of birth and language spoken at home), educational attainment, geographic remoteness, and SES. Educational achievement categorised respondents into one of the three groups (secondary school, technical and further education [TAFE], tertiary). SES was based on participant postcode and categorised respondents into one of the three groups (low, middle, and high SES) depending on their score on the socioeconomic indexes for areas index of relative socioeconomic disadvantage [25]. Clinical data included cancer type, time since diagnosis, treatment types (surgery, chemotherapy, radiotherapy, immunotherapy, or other), and BMI (kilogrammes/metres^2^).

#### Internet access, usage, and content

Internet access and usage frequency were measured using a modified version of the internet use survey from the Finding My Way Advanced study [26]. Two items measuring internet access were added to the existing survey (i.e., “Where do you usually access the internet for personal use?”). Respondents indicated the frequency of their internet use on a 7-item Likert scale, with options ranging from 1 = *I do not use the internet* to 7 = *[I use the internet] multiple times a day*.

#### Self-efficacy

Self-efficacy was measured using the 10-item General Self-Efficacy Scale (GSE) [27]. The scale measures one’s belief that they can overcome problems. Each item has 4 response options: 1 = *not true*, 2 = *hardly true*, 3 = *moderately true*, and 4 = *exactly true.* Scores range from 10 to 40 with higher scores indicating greater self-efficacy. In an analysis across 25 countries, the GSE demonstrated acceptable internal consistency (*α* = 0.91) [28]. In the current study, the scale demonstrated acceptable internal consistency (α = 0.89).

#### Health literacy

Health literacy was measured using three subscales of the Health Literacy Questionnaire (HLQ) [29]: (i) *actively managing my health*, (ii) *ability to find good health information*, and (iii) *understand health information enough to know what to do*. The “actively managing my health” scale contains four items (e.g., “I have all the information I need to look after my health”) with four response options: *strongly disagree*, *disagree*, *agree*, *and strongly agree.* The “ability to find good health information” (e.g., “Find information about health problems”) and “understand health information” (e.g., “accurately follow instructions from health providers”) subscales contain five items each with five response options: *cannot do*, *very difficult*, *difficult*, *quite easy*, *and very easy*. Higher scores equal higher levels of health literacy in that domain. The HLQ has previously demonstrated acceptable reliability (range between 0.86 and 0.89) [29]. In our study, all three scales demonstrated acceptable reliability (actively managing my health (*α* = 0.84), ability to find good health information (*α* = 0.89), understand health information enough to know what to do (*α* = 0.88)).

#### Social support

Social support was measured using the Social Support Survey developed for the Medical Outcomes Study (MOS-SSS) [30]. The survey yields a total social support score across 21 items, and full sub-scale scores (perceived emotional/informational, tangible, affectionate, and positive social interaction). Only the full-scale score was used in the current study. The MOS-SSS has been used in other cancer survivor research [31, 32]. The scale has demonstrated high reliability, with reported Cronbach’s alpha coefficients for the sub-scales and total scale score ranging from 0.91 to 0.97 [30]. In the current study, the overall scale demonstrated acceptable reliability (*α* = 0.97).

#### Delivery modality preference

The outcome variable of delivery modality preference was measured in three ways. A dichotomous outcome measure asked participants to indicate their delivery modality preference for a healthy lifestyle after cancer program as either *telephone* or *online*. Two secondary continuous modality measures were developed that asked participants to rate the *likelihood* of using a healthy lifestyle after cancer program delivered via (1) telephone or (2) an internet platform. Response options were on a 6-point Likert scale ranging from 1 = *extremely likely* to 6 = *extremely unlikely*. Each of these items was considered separately.

### Data analysis

Analyses for this study were performed using the IBM SPSS statistics software version 25. Descriptive statistics summarised participants’ sociodemographic profile, clinical characteristics, and internet use. Chi-square analyses and independent sample *t*-tests were conducted to compare (a) the preference groups and (b) survey completers and non-completers. Differences between groups were considered significant if *p* < 0.05. Due to the small sample size, expectation maximisation was used to impute the missing values for the continuous measures, including BMI, the GSE, the HLQ scales, and the MOS-SSS. Missing item imputation was not conducted for delivery modality preference, as categorical imputation methods were deemed inappropriate.

To test for whether SCT factors mediated the relationship between sociodemographic and clinical characteristics and modality preference, PROCESS [33] was used to conduct a series of multiple logistic regression and multiple linear regression analyses. Prior to analyses, the assumptions for ordinary least square regressions were tested. The assumption of normality of error was met as assessed by visual inspection of a Q-Q plot. There was no evidence of heteroscedasticity as indicated by the Breusch-Pagan test. However, there was evidence of multicollinearity, as *actively managing health* was corrected with *reading and understanding health information* (*r*(168) = 0.77, *p* =  < 0.001) and self-efficacy (*r*(168) = 0.42, *p* =  < 0.001). This variable was consequently removed, and there was no other evidence of multicollinearity as assessed by tolerance values greater than 0.1.

To test the model using the dichotomous outcome, five multiple logistic regressions were conducted using a parallel multiple mediator model. In each analysis, one sociodemographic variable (i.e., either age, gender, SES, BMI, and education) was entered as the independent variable and the remaining sociodemographic variables were entered as covariates. The SCT factors of self-efficacy, social support, and health literacy (two subscales) were entered into each analysis as mediators. For each direct and indirect association, bootstrap confidence intervals were based on 10,000 resamples and considered significant if the upper and lower limit did not include zero.

To determine whether modality preference varied between the dichotomous and continuous outcome measure, analyses testing the model were repeated using the two continuous outcome measures for delivery modality preference. Thus, ten multiple linear regressions were conducted, five with continuous-telephone and five with continuous-internet measures as the outcome.

## Results

### Demographic profile

One hundred ninety-two people responded to the study advertisements, with a total of 168 respondents completing the survey. Demographic and clinical characteristics are presented in Table 1 by modality group and overall sample. Most participants were female (91.9%), had been diagnosed with breast cancer (79.7%), were tertiary educated (49.7%), and were in the high percentile for SES (45.2%). Most participants used the internet multiple times a day (78.3%) and accessed the internet at home (98.0%). In addition, 38.3% of participants had accessed a healthy lifestyle program in the past and had primarily accessed the program via internet (40.6%) or face-to-face delivery (45.3%).

**Table 1:** Participant demographic and clinical characteristics

Characteristic	Non-responders for preference (*N* = 19)	Telephone (*N* = 40)	Internet (*N* = 109)	Overall sample (*N* = 168)	Group differences^†^
	*M* (*SD*)	*M* (*SD*)	*M* (*SD*)	*M* (*SD*)	*p*
Age	49.32 (19.1)	61.7 (7.6)	62.1 (10.6)	60.7(11.7)	.81
BMI	26.37 (4.9)	27.1 (5.0)	26.0 (4.5)	26.4 (4.9)	.23
Age at diagnosis	53.33 (5.1)	54.6 (9.7)	52.2 (10.9)	52.9 (10.5)	.24
Time since diagnosis	7.6 (4.5)	6.8 (5.54)	9.85 (8.20)	9.02(7.63)	.03*
	*n* (%)	*n* (%)	*n* (%)	*n* (%)	*p*
Gender	(*n* = 11)	(*n* = 40)	(*n* = 109)	(*n* = 160)	n/a
Female	1 (5.3)	37 (92.5)	100 (91.7)	147 (91.9)	
Male	10 (52.6)	3 (7.5)	9 (8.3)	13 (8.1)	
Aboriginal or Torres Strait Islander	(*n* = 11)	(*n* = 40)	(*n* = 109)	(*n* = 149)	.53
Aboriginal	-	-	2 (1.80)	2 (1.1)	
Neither	11 (57.9)	39 (97.5)	106 (97.2)	145 (97.3)	
Prefer not to say	-	1 (2.5)	1 (0.9)	2 (1.3)	
Educational achievement	(*n* = 8)	(*n* = 40)	(*n* = 109)	(*n* = 157)	.82
Secondary school	2 (10.5)	10 (25.0)	22 (20.2)	34 (21.7)	
TAFE	2 (10.5)	11 (27.5)	32 (29.4)	45 (28.7)	
Tertiary	4 (21.1)	19 (47.5)	55 (50.5)	78 (49.7)	
SES	(*n* = 8)	(*n* = 39)	(*n* = 108)	(*n* = 155)	.11
Low	1 (5.3)	15 (38.5)	23 (21.3)	39 (25.2)	
Middle	1 (5.3)	10 (25.6)	35 (32.4)	46 (29.7)	
High	6 (31.6)	14 (35.9)	50 (46.3)	70 (45.2)	
Country of birth	(*n* = 8)	(*n* = 40)	(*n* = 109)	(*n* = 157)	.19
Australia	6 (31.6)	36 (90.0)	84 (77.1)	126 (75.0)	
Europe	2 (10.6)	3 (7.5)	16 (14.6)	21 (13.20)	
Other^a^	-	1 (2.5)	9 (8.3)	10 (6.0)	
Characteristic	Non-responders for preference (*N* = 19)	Telephone (*N* = 40)	Internet (*N* = 109)	Overall sample (*N* = 168)	Group differences†
	*n* (%)	*n* (%)	*n* (%)	*n* (%)	*p*
Cancer type	(*n* = 4)	(*n* = 39)	(*n* = 109)	(*n* = 153)	n/a
Breast	3 (15.8)	31 (77.5)	88 (80.7)	122 (79.7)	
Colorectal	-	-	2 (1.8)	2 (1.3)	
Head and neck	-	1 (2.5)	1 (0.9)	2 (1.3)	
Lymphoma	-	2 (5.0)	2 (1.8)	4 (2.6)	
Lung	-	1 (2.5)	-	1 (0.7)	
Ovarian	-	1 (2.5)	1 (0.9)	2 (1.3)	
Prostate	-	2 (5.0)	5 (4.6)	7 (4.6)	
Other^b^	1 (5.3)	2 (5.0)	10 (9.2)	13 (8.5)	
Completed treatment		(*n* = 40)	(*n* = 108)	(*n* = 161)	.07
Yes	7 (36.8)	28 (70.0)	92 (85.2)	127 (78.9)	
No	4 (21.1)	10 (25.0)	11 (10.2)	25 (15.5)	
Unsure	2 (5.0)	2 (5.0)	5 (4.6)	9 (5.6)	
Treatment received^c^	(*n* = 11)	(*n* = 40)	(*n* = 109)	(*n* = 153)	
Surgery	3 (15.8)	38 (95.0)	105 (96.3)	146 (95.4)	.71
Chemotherapy	3 (15.8)	24 (60.0)	73 (67.0)	100 (65.4)	.43
Radiotherapy	3 (15.8)	25 (62.5)	74 (67.9)	102 (66.7)	.54
Immunotherapy	-	2 (5.0)	14 (12.8)	16 (10.5)	.17
Other	2 (10.6)	10 (25.0)	26 (23.9)	38 (22.8)	.39

^†^Group differences between participants who indicated preference for telephone or internet delivery

^*^p < .05; n/a indicates that assumptions were violated to conduct a chi-square analysis (i.e., 25% of cell counts < 5)

^a^Africa (*n* = 4), Asia (*n* = 3), New Zealand (*n* = 2), and North America (*n* = 1)

^b^Melanoma (*n* = 3), bile duct (*n* = 1), Ewing’s (*n* = 1), GIST (*n* = 1), Langerhans histiocytosis X (*n* = 1), myeloma (*n* = 1), oesophagus (*n* = 2), primary peritoneal cancer (*n* = 1), bowel (*n* = 1), and thyroid (*n* = 1)

^c^Multiple responses allowed

### Modality preference

A greater proportion of participants preferred the internet modality (*n* = 109; 64.9%) in comparison to telephone (*n* = 40; 23.8%) (*X*^2^ (1, 149) = 32.0,* p* < 0.001). Nineteen (11.3%) respondents did not complete the item. Time since diagnosis significantly differed between modality groups, with telephone modality preferred by those with a shorter time since diagnosis (*M* = 6.80, *SD* = 5.54) compared to those who preferred the internet modality (*M* = 9.85, *SD* = 8.20) (*t* (146) =  − 2.15, *p* = 0.03). Those who did not report a modality preference were younger (*M* = 49.3, *SD* = 19.1) than those who reported a modality preference (*M* = 62.0, *SD* = 9.8) (*t* (17.0) =  − 2.7, *p* = 0.01). Table 2 summarises the sample’s internet access and usage.

**Table 2:** Description of sample internet use

Variable	Telephone (*n* = 40)	Internet (*n* = 109)	Overall sample (*N* = 168)	Group differences
	*n* (%)	*n* (%)	*n* (%)	*p*
Frequency of internet use	(*n* = 40)	(*n* = 109)	(*n* = 152)	n/a
Multiple times a day	27 (67.5)	90 (82.6)	119 (78.3)	
Once a day	4 (10.0)	13 (11.9)	17 (11.2)	
A few times a week	5 (12.5)	5 (4.6)	10 (6.6)	
Once a week	1 (2.5)	-	1 (0.7)	
Less than fortnightly	2 (5.0)	1 (0.9)	3 (2.0)	
I do not use the internet	1 (2.5)	-	2 (1.3)	
Where internet is accessed^a^	(*n* = 39)	(*n* = 109)	(*n* = 150)	
Home	38 (95.0)	107 (98.2)	147 (98.0)	n/a
Work	7 (17.9)	25 (22.9)	32 (21.3)	.52
Mobile data	3 (7.50)	4 (3.60)	7 (1.2)	n/a
Accessed a healthy lifestyle program before	(*n* = 40)	(*n* = 109)	(*n* = 151)	n/a
Yes	20 (50.0)	45 (41.3)	66 (43.7)	
No	18 (45.0)	60 (55.0)	79 (52.3)	
Unsure	2 (5.0)	4 (3.7)	6 (4.0)	
Modality of program accessed before	(*n* = 19)	(*n* = 44)	(*n* = 64)	.02*
Via telephone	5 (26.3)	4 (9.1)	9 (14.1)	
Via internet	3 (15.8)	23 (52.3)	26 (40.6)	
Via face-to-face	11 (57.9)	17 (38.6)	29 (45.3)	

^a^Multiple responses are allowed; n/a indicates that assumptions were violated to conduct a chi-square analysis (i.e., 25% of cell counts < 5). The total of participants in the modality groups does not equate to the overall sample due to 19 participants not completing the dichotomous measure of delivery modality preference

### SCT mediation testing

Table 3 summarises the regression coefficients of the direct association between the sociodemographic factors (IV) and the SCT factors (MV). With respect to the first part of the mediation chain (IV→MV), age was positively, weakly associated with social support. Gender, BMI, and SES did not have a significant relationship with any SCT factors. Participants who had completed TAFE (*M* = 4.15, *SD* = 0.58) scored significantly higher on *finding good health information* compared to those who completed secondary education or below (*M* = 4.16, *SD* = 0.59). Participants who completed tertiary education (*M* = 4.35, *SD* = 0.56) scored significantly higher on *understanding health information well enough to know what to do* in comparison to those who completed secondary education or below (*M* = 4.02, *SD* = 0.52).

**Table 3:** Model coefficients (direct associations) for the regression analysis of the association between sociodemographic factors on social-cognitive factors

Sociodemographic factor								
	Self-efficacy		Find good health information		Understanding health information		Social support	
	*B* [95% CI]	*p*	*B* [95% CI]	*p*	*B* [95% CI]	*p*	*B* [95% CI]	*p*
Age	0.05 [− 0.03, 0.12]	.20	0.004 [− 0.01, 0.02	.45	< 0.001 [− 0.01,0.01]	.95	0.02 [0.004, 0.03]	.01*
Gender	− 0.73 [− 3.24, 1.78]	.57	0.11 [− 0.27, 0.48]	.58	0.10 [− 0.24, 0.44]	.56	− 0.33 [− 0.81, 0.17]	.19
Middle SES^a^	1.24 [− 0.54, 3.01]	.17	0.15 [− 0.11, 0.43]	.25	0.05 [− 0.19, 0.29]	.66	0.13 [− 0.22, 0.47]	.46
High SES^a^	0.15 [− 1.58, 1.87]	.87	0.14 [− 0.12, 0.40]	.29	0.10 [− 0.13, 0.33]	.40	0.15 [− 0.18, 0.47]	.37
BMI	− 0.08 [− 0.23, 0.07]	.31	0.01 [− 0.02, 0.03]	.70	0.001[− 0.02, 0.02]	.94	− 0.02 [− 0.05, 0.01]	.19
TAFE^b^	0.36 [− 1.57, 2.29]	.71	0.35 [0.06, 0.65]	.02*	0.19 [− 0.04, 0.02]	.14	0.23 [− 0.14,0.61]	.22
Tertiary^b^	0.63 [− 1.20, 2.47]	.50	0.26 [0.02, 0.54]	.07	0.32 [0.07, 0.57]	.01*	0.25 [− 0.10, 0.61]	.16
Constant	30.77 [22.39, 39.14]	< .001**	3.12 [1.86, 4.39]	< .001**	3.77 [2.64, 4.90]	< .001**	3.69 [2.06, 5.31]	< .001**

*df* = 7, 136. *N* = 147. *B* is the unstandardized coefficient

*p < .05; **p < .001

Reference levels: ^a^low SES

^b^Secondary

The multiple regression models that included the combined association between all the sociodemographic factors with self-efficacy (*F* (7, 139) = 0.74, *p* = 0.64,* R*^2^ = 0.04) and health literacy (*ability to find good health information*: *F* (7, 139) = 1.29, *p* = 0.64, *R*^2^ = 0.04; *understanding health information well enough to know what to do*: *F* (7, 139) = 1.29, *p* = 0.26,* R*^2^ = 0.06) were non-significant. However, the multiple regression model of all the sociodemographic factors combined statistically significantly explained 10% of the variance in social support (*F* (7, 139) = 2.23, *p* = 0.04,* R*^2^ = 0.10).

With respect to the second and third parts of the mediation chain (IV →DV; MV → DV), no sociodemographic or SCT factor separately or in combination had a significant direct (Table 4) or indirect association with delivery modality preference (see Online Resource 1).

**Table 4:** Model coefficients (direct associations) of sociodemographic factors and social cognitive factors on delivery modality preference

Variable	*B*	95% confidence intervals (*B*)	*p*
Socio-demographic factors → delivery modality preference			
Age	0.003	[− 0.04, 0.05]	.89
Gender	− 0.05	[− 1.49, 1.40]	.95
Middle SES^a^	0.79	[− 0.19, 1.77]	.13
High SES^a^	0.79	[− 0.15, 1.73]	.10
BMI	− 0.06	[− 0.15, 1.73]	.10
TAFE^b^	0.14	[− 0.97, 1.24]	.81
Tertiary^b^	0.05	[− 0.98, 1.07]	.93
Mediators → delivery modality preference			
Self-efficacy	− 0.01	[− 0.12, 0.10]	.86
Ability to find good health information	0.85	[− 1.81, 0.40]	.21
Understand health information well enough to know what to do	− 0.71	[− 1.71, 0.50]	.28
Social support	0.14	[− 0.37, 0.66]	.58
Constant	1.26	[− 4.45, 6.97]	.67

*df* = 11, 136. *N* = 147. *B* is the unstandardized coefficient

Reference levels: ^a^low SES; ^b^secondary

Comparable direct associations between the sociodemographic factors on the social-cognitive factors were observed when utilising the continuous measure for telephone preference. One difference that emerged was the direct association between gender and the telephone preference, whereby females (*M* = 3.62, *SD* = 1.50) reported a greater likelihood in using a telephone-delivered healthy living program in comparison to males (*M* = 2.36, *SD* = 1.12) (*B* = 1.35, *p* = 0.02). No significant indirect associations between the sociodemographic factors and delivery modality preference through the SCT factors were found (see Online Resource 2).

The model using a continuous outcome for likelihood of using internet delivery did not have any comparable differences to the dichotomous-outcome model. No direct associations between the sociodemographic factors and delivery modality preference or indirect associations through the social-cognitive factors were observed (see Online Resource 2).

## Discussion

In this study of survivors’ preferences for accessible delivery of a healthy living intervention, nearly two thirds of participants preferred internet over telephone delivery. This finding is consistent with Phillips et al. which found that 69.5% of their breast cancer survivor participants indicated that they would use a website or app to help increase their physical activity [20]. Although these data offer some insight into *which* modality cancer survivors prefer for a healthy living intervention, factors related to *why* they might have this preference remain unclear. Contrary to the findings of previous studies [20], we did not find a relationship between age, SES, BMI, or educational attainment, with preference for telephone or internet delivery using either dichotomous or continuous measure. Neither gender particularly endorsed a *preference* for the telephone delivery; however, females reported being more *likely* to use this modality in comparison to their male counterparts. These findings suggest that sociodemographic factors may not play a strong role in delivery modality preference and internet delivery should be considered when developing an accessible health intervention, especially for the breast cancer survivor population. The inconsistency between the current study and previous reports on the relationship between sociodemographic factors and finding health information on the internet may be attributed to an increase in digital inclusion in the Australian population. Since 2014, the Australian Digital Inclusion Index has recorded a steady increase in digital access and affordability for Australians, even in those typically described as internet novices, including older adults, indigenous Australians, individuals with a disability, and those with a lower SES [34, 35]. Therefore, differences in preference could be attributed to *how* cancer survivors use the internet, rather than *if* they access the internet. Indeed, participants in our study who reported an interest in the internet delivery also tended to report accessing the internet multiple times a day. Future research should consider using models that relate to technology usage, for example, the technology acceptance model [36], to explore this relationship further.

To our knowledge, this is the first study to investigate the influence of SCT factors, specifically self-efficacy, health literacy, and social support, on cancer survivor’s delivery preference for a health behaviour intervention. These SCT factors were investigated as they have previously demonstrated a positive relationship with cancer survivors using alternative methods (i.e., the internet) other than their physician to seek health information, and it was posited that this may be indicative of a preference for internet delivery of health behaviour interventions. However, the current study demonstrated no evidence of a relationship between these SCT factors and delivery modality preference. Consequently, these SCT factors may not impact delivery modality preference but instead may impact on delivery modality access and adherence. Lower levels of health literacy and social support have been identified as two barriers to the uptake and engagement with digital health interventions [37, 38]. Although there is currently limited evidence supporting the impact of self-efficacy on uptake and use of digital health interventions [39], SCT suggests that individuals with lower self-efficacy tend to make half-hearted attempts at behaviour change and would benefit from the additional support that is offered by the telephone delivery modality [22]. Thus, these SCT factors remain an important consideration for the development of health interventions for cancer survivors.

One unexpected finding in the current study was that cancer survivors who preferred internet delivery were further into their survivorship trajectory than those who preferred the telephone delivery. This finding may reflect how needs change throughout long-term cancer survivorship. Up until the 5-year post-treatment mark, cancer survivors may still be experiencing many of the physical and social consequences of cancer [40] and prefer the additional and personalised support offered by the telephone modality. As they progress into long-term survivorship, they may have less engagement with health services and no longer receive such support [41]. Further, most survivorship interventions implemented in research settings have focused on the 5-year period following cancer diagnosis. However, research has demonstrated that long-term cancer survivors can benefit from health interventions [42]. Future health interventions supporting cancer survivors should consider opening their eligibility to include those who are further along in their cancer survivorship.

Three limitations that should be reflected on when reviewing the current study include the timing of this study in relation to the COVID-19 pandemic, the small sample size, and the measurement of preferences. Data collection for the current study occurred 6 months prior to the introduction of social distancing restrictions implemented during the COVID-19 pandemic. These restrictions reduced health practitioners’ ability to address health concern in face-to-face appointments and resulted in a rapid implementation of telemedicine (e.g., appointments delivered over the phone or via teleconferencing) [6]. Digital inclusion and uptake of the internet may have been further accelerated following the COVID-19 pandemic, as people spent more time online for work, school, and social connection. It is unclear how this might influence preferences for accessing an accessible health intervention. Preference for the internet modality may increase, due to greater familiarity with the internet and digital health modalities. However, emerging research in ongoing delivery of telemedicine has been mixed [43–45], with some studies finding that patients wish to return to face-to-face appointments following extended use of telemedicine [45]. It would be beneficial to replicate the current study to observe how preferences for accessible delivery modalities might change following the COVID-19 pandemic. Another limitation of this study is the small sample size, particularly the telephone preference group, which did not reach the targeted sample of 80. Consequently, the current may not have had enough power to detect the association of socio-demographic and SCT factors on delivery modality preference. Finally, the current study utilised a non-validated measure to capture delivery modality preferences. This is a common limitation in this area of research, as there is currently no validated measure for delivery modality preference. Consequently, each study has developed their own measure for their individual project [15, 18], and this inconsistency may impede our ability to find consistent results. Future research should consider developing and validating a measure for delivery modality preference to increase consistency across studies in this area.

## Conclusion

Delivery modality has been a largely overlooked area of research when considering cancer survivors’ preferences for health behaviour interventions. The cancer survivors in the current study preferred internet delivery to telephone. To our knowledge, this is the first study to investigate and rule out the contribution of SCT factors to cancer survivor’s delivery preferences. Instead, user’s stage of cancer survivorship should be considered in the development of a health behaviour program. Individuals who are further along in their survivorship trajectory are largely overlooked when offering health interventions, and an internet modality may be uniquely suited to meet their needs. How replicable these findings are since the advent of COVID-19 is an avenue for future research.

## Supplementary Information

Below is the link to the electronic supplementary material.

Supplementary file1 (DOCX 25 KB)

Supplementary file2 (DOCX 63 KB)

## Data Availability

The datasets generated and/or analysed during the current study are available from the corresponding author on reasonable request.
