# Supplementary material for: Modality preferences for health behaviour interventions for post-treatment cancer survivors: a theoretical investigation
Source: Support Care Cancer. 2023 Feb 2;31(2):143. doi: 10.1007/s00520-023-07607-8 (PMC9892669; doi:10.1007/s00520-023-07607-8)
Supplement: Supplementary file 1 — Supplementary file1 (DOCX 25 KB) [file 520_2023_7607_MOESM1_ESM.docx]

**Modality preferences for health behaviour interventions for post-treatment cancer survivors: a theoretical investigation.**

Supportive Care in Cancer

Morgan Leske, Bogda Koczwara, Julia Morris, and Lisa Beatty

Corresponding author: Morgan Leske

Affiliation: College of Education, Psychology, and Social Work, Flinders University, Adelaide, SA, Australia

Email address: [morgan.leske@flinders.edu.au](mailto:morgan.leske@flinders.edu.au)

**Table 5.**

*Model Coefficients for the Indirect associations between Sociodemographic Factors on Delivery Modality Preference.*

|  | Social Cognitive Factors | | | | | | | | | |
| --- | --- | --- | --- | --- | --- | --- | --- | --- | --- | --- |
| Sociodemographic Factor | Self-efficacy | | Finding good health information | | Understanding health information | | Social Support | | Total | |
|  | B | [95% CI] | B | [95% CI] | B | [95% CI] | B | [95% CI] | B | [95% CI] |
| Age | -0.0005 | [-0.01, 0.01] | 0.003 | [-0.01, 0.02] | <0.001 | [-0.01, 0.01] | 0.003 | [-0.01, 0.02] | 0.01 | [-0.01, 0.02] |
| Gender | 0.01 | [-0.16, 0.24] | 0.08 | [-0.33, 0.55] | -0.07 | [-0.53, 0.24] | -0.05 | [-0.31, 0.19] | -0.03 | [-0.47, 0.40] |
| Middle SES^a^ | 0.003 | [-0.26, 0.29] | 0.11 | [-0.18, 0.55] | -0.03 | [-0.34, 0.19] | 0.02 | [-0.10, 0.24] | - | - |
| High SES^a^ | 0.001 | [-0.18, 0.17] | 0.09 | [-0.19, 0.55] | -0.05 | [-0.39, 0.20] | 0.02 | [-0.10, 0.25] | - | - |
| BMI | 0.001 | [-0.02, 0.02] | .004 | [-0.02, 0.03] | -0.001 | [-0.03, 0.02] | 0.003 | [-0.03, 0.01] | 0.01 | [-0.03, 0.01] |
| TAFE^b^ | -0.003 | [-0.13, 0.19] | 0.32 | [-0.15, 1.08] | -0.13 | [-0.69, 0.17] | 0.04 | [-0.14, 0.31] | - | - |
| Tertiary^b^ | -0.01 | [-0.16, 0.21] | 0.24 | [-0.12, 0.92] | -0.21 | [-0.97, 0.27] | 0.04 | [-0.14,  0.29] | - | - |

Note: N=147, B is the unstandardized coefficient.

Reference levels: ^a^Low SES; ^b^Secondary.

-Indirect effects are not calculated for multinomial variables in PROCESS
