# Supplementary material for: Modality preferences for health behaviour interventions for post-treatment cancer survivors: a theoretical investigation
Source: Support Care Cancer. 2023 Feb 2;31(2):143. doi: 10.1007/s00520-023-07607-8 (PMC9892669; doi:10.1007/s00520-023-07607-8)
Supplement: Supplementary file 2 — Supplementary file2 (DOCX 63 KB) [file 520_2023_7607_MOESM2_ESM.docx]

**Modality preferences for health behaviour interventions for post-treatment cancer survivors: a theoretical investigation.**

Supportive Care in Cancer

Morgan Leske, Bogda Koczwara, Julia Morris, and Lisa Beatty

Corresponding author: Morgan Leske

Affiliation: College of Education, Psychology, and Social Work, Flinders University, Adelaide, SA, Australia

Email address: [morgan.leske@flinders.edu.au](mailto:morgan.leske@flinders.edu.au)

**Mediation Testing: Continuous Outcome (Telephone Continuous Scale).**

**Table 6.**

*Model Coefficients for the Direct associations between the Sociodemographic Factors and Social Cognitive Factors (Telephone Continuous scale*)*.*

| Sociodemographic  Factor | Social Cognitive Factor | | | | | | | |
| --- | --- | --- | --- | --- | --- | --- | --- | --- |
|  | Self-efficacy | | Find good health information | | Understanding health information | | Social Support | |
|  | B [95% CI] | *p* | B [95% CI] | *p* | B [95% CI] | *p* | B [95% CI] | *p* |
| Age | 0.04 [-0.03, 0.11] | .25 | 0.004 [-0.01, 0.02] | .48 | -0.0002 [-0.01, 0.01] | .96 | 0.02 [0.004, 0.03] | .01* |
| Gender | -0.29 [-2.84, 2.26] | .82 | 0.18 [-0.21, 0.56] | .36 | 0.08 [-0.26, 0.42] | .64 | -0.38 [-0.88, 0.12] | .13 |
| Middle SES^a^ | 1.20 [-0.56, 2.95] | .18 | 0.15 [-0.11, 0.41] | .26 | 0.05 [-0.18, 0.29] | .66 | 0.13 [-0.21, 0.48] | .45 |
| High SES^a^ | 0.02 [-1.70, 1.73] | .99 | 0.11 [-0.14, 0.37] | .38 | 0.09 [-0.14, 0.32] | .46 | 0.18 [-0.16, 0.51] | .30 |
| BMI | -0.07 [-0.22, 0.07] | .33 | 0.005 [-0.02, 0.03] | .67 | 0.002 [-0.02, 0.02] | .85 | -0.02 [-0.05, 0.01] | .15 |
| TAFE^b^ | 0.75 [-1.17, 2.67] | .44 | 0.39 [0.11, 0.68] | .01* | 0.24 [0.02, 0.50] | .07 | 0.23 [-0.15, 0.60] | .24 |
| Tertiary^b^ | 0.85 [-0.98, 2.70 | .36 | 0.31 [0.03, 0.58] | .03* | 0.36 [0.12, 0.61] | .004* | 0.23 [-0.13, 0.59] | .21 |
| Constant | 29.94 [17.41, 36.39] | <.001** | 2.97 [1.72, 4.23] | <.001** | 3.77 [2.65, 4.89] | <.001** | 3.84 [2.21, 5.48] | .001* |
| Note: *df* =10, 136. N=147. B is the unstandardized coefficient. Reference levels: ^a^Low SES; ^b^Secondary; * *p*< .05; ** *p*< .001. | | | | | | | | |

Table 7.

*Model Coefficients (Direct associations) of Sociodemographic Factors and Social Cognitive Factors Interest in Telephone Delivery.*

| Variable | B | 95% confidence intervals (B) | *p* |
| --- | --- | --- | --- |
| Socio-demographic factors →  Interest in telephone modality  Age | -0.002 | [-0.03, 0.03] | .90 |
| Gender | 1.28 | [0.32, 2.25] | .01* |
| Middle SES^a^ | 0.34 | [-0.32, 1.00] | .32 |
| High SES^a^ | 0.37 | [-0.27, 1.01] | .25 |
| BMI | -0.01 | [-0.07, 0.05] | .79 |
| TAFE^b^ | -0.34 | [-1.08, 0.39] | .35 |
| Tertiary^b^ | -0.08 | [-0.79, 0.62] | .81 |
| Social Cognitive Factors → Interest in telephone modality  Self-efficacy | 0.04 | [-0.04, 0.11] | .31 |
| Ability to find good health information | 0.43 | [-0.25, 1.11] | .21 |
| Understand health information well enough to know what to do | -0.60 | [-1.34, 0.16] | .12 |
| Social support | 0.08 | [-0.27, 0.42] | .67 |
| Constant | 0.54 | [-3.27, 4.35 ] | .78 |

Note: *df* =10, 136. N=147. B is the unstandardized coefficient. Reference levels: ^a^Low SES; ^b^Secondary; **p*< .05.

Table 8.

*Model Coefficients for the indirect associations between Sociodemographic Factors on interest in the telephone delivery modality*

| Social Cognitive Factor | | | | | | | | | | | | |
| --- | --- | --- | --- | --- | --- | --- | --- | --- | --- | --- | --- | --- |
| Sociodemographic  Factor | Self-efficacy | | Finding good health information | | Understanding health information | | Social Support | | Total | | |  |
|  | B | [95% CI] | B | [95% CI] | B | [95% CI] | B | [95% CI] | B | [95% CI] |  |  |
| Age | 0.005 | [-0.01, 0.02] | 0.002 | [-0.01, 0.01] | 0.001 | [-0.01,0.01] | 0.001 | [-0.01, 0.01] | 0.01 | [-0.01, 0.02] |  |  |
| Gender | -0.01 | [-0.11, 0.11] | 0.08 | [-0.14, 0.35] | -0.05 | [-0.28, 0.14] | -0.03 | [-0.23, 0.11] | 0.004 | [-0.28, 0.27] |  |  |
| Middle SES^a^ | 0.06 | [-0.07, 0.23] | 0.06 | [-0.08, 0.33] | -0.04 | [-0.26, 0.13] | 0.01 | [-0.05, 0.08] | - | - |  |  |
| High SES^a^ | 0.01 | [-0.12, 0.12] | 0.05 | [-0.10, 0.30] | -0.06 | [-0.30, 0.12] | 0.01 | [-0.05, 0.08] | - | - |  |  |
| BMI | -0.003 | [-0.02, 0.01] | .002 | [-0.01, 0.02] | -0.001 | [-0.02, 0.01] | -0.002 | [-0.01, 0.01] | 0.003 | [-0.02, 0.01] |  |  |
| TAFE^b^ | -0.03 | [-0.05, 0.18] | 0.17 | [-0.13, 0.53] | -0.14 | [-0.43, 0.06] | 0.02 | [-0.08, 0.17] | - | - |  |  |
| Tertiary^b^ | -0.03 | [-0.06, 0.19] | 0.14 | [-0.09, 0.49] | -0.21 | [0.60, 0.07] | 0.02 | [-0.08, 0.14] | - | - |  |  |

Note: N=147, B is the unstandardized coefficient.

Reference levels: ^a^Low SES; ^b^Secondary.

-Indirect effects are not calculated for multinomial variables in PROCESS.

**Mediation Testing: Continuous Outcome (Internet Continuous Scale).**

Table 9.

*Model Coefficients (Direct associations) for the Regression Analysis of associations between Sociodemographic Factors and Social-Cognitive Factor (Internet*

*Continuous Scale*)

| Sociodemographic  Factor |  | | Social Cognitive Factor | |  | |  | |
| --- | --- | --- | --- | --- | --- | --- | --- | --- |
|  | Self-efficacy | | Finding good health information | | Understanding health information | | Social Support | |
|  | B [95% CI] | *p* | B [95% CI] | *p* | B [95% CI] | *p* | B[95% CI] | *p* |
| Age | 0.05 [-0.03, 0.12] | .25 | 0.001 [-0.01, 0.02] | .44 | -0.001[-0.01, 0.01] | .98 | 0.02 [0.002,0.03] | .01* |
| Gender | -0.96 [-3.46, 1.55] | .45 | 0.08 [-0.30, 0.45] | .68 | 0.003 [-0.33, 0.34] | .98 | -0.34 [-0.82, 0.15] | .17 |
| Middle SES^a^ | 1.23 [-0.55, 3.02] | .17 | 0.16 [-0.11, 0.42] | .24 | 0.06 [-0.20, 0.30] | .64 | 0.13 [-0.22, 0.47] | .46 |
| High SES^a^ | 0.21 [-1.45, 2.11] | .81 | 0.14 [-0.12, 0.40] | .28 | 0.11 [-0.12, 0.34] | .35 | 0.16 [-0.17, 0.50] | .33 |
| BMI | -0.08[-0.23, 0.07] | .32 | 0.01[-0.02, 0.03] | .69 | 0.002 [-0.02, 0.02] | .87 | -0.02[-0.05, 0.01] | .16 |
| TAFE^b^ | 0.50 [-1.43, 2.42] | .61 | 0.36 [0.07, 0.65] | .02* | 0.21 [-0.11, 0.45] | .11 | 0.24 [-0.13, 0.62] | .20 |
| Tertiary^b^ | 0.55 [-1.30, 2.38] | .55 | 0.26 [-0.01, 0.54] | .06 | 0.33 [0.08, 0.57] | .01* | 0.25 [-0.11, 0.61] | .14 |
| Constant | 31.27 [22.93, 39.62] | <.001** | 3.17 [1.92, 4.42] | <.001 ** | 3.91 [2.80, 5.04] | <.001** | 3.75 [2.13, 5.37] | <.001** |
| Note: *df* =10, 148. N=147. B is the unstandardized coefficient. Reference levels: ^a^Low SES; ^b^Secondary; * *p*< .05; ***p*<.001. | | | | | | | | |

Table 10.

*Model Coefficients (Direct associations) of Sociodemographic Factors and Social Cognitive Factors on Interest in Internet delivery.*

| Variable | B | 95% confidence intervals (B) | *p* |
| --- | --- | --- | --- |
| Sociodemographic factors →  Delivery Modality Preference  Age | 0.02 | [-0.002, 0.04] | .07 |
| Gender | 0.23 | [-0.57, 1.23] | .57 |
| Middle SES^a^ | -0.31 | [-0.88, 0.26] | .28 |
| High SES^a^ | -0.25 | [-0.80, 0.30] | .37 |
| BMI | -0.02 | [-0.06, 0.03] | .54 |
| TAFE^b^ | -0.27 | [-0.90, 0.36] | .40 |
| Tertiary^b^ | -0.40 | [-1.00, 0.21] | .20 |
| Social Cognitive Factors →  Delivery Modality Preference  Self-efficacy | 0.04 | [-0.02, 0.11] | .19 |
| Ability to find good health information | -0.23 | [-0.81, 0.36] | .44 |
| Understand health information well enough to know what to do | 0.08 | [-0.56, 0.73] | .25 |
| Social support | -0.05 | [-0.34, 0.25] | .74 |
| Constant | 1.36 | [-1.94, 4.66] | .42 |

Note: *df* =10, 136. N=147. B is the unstandardized coefficient. Reference levels: ^a^Low SES; ^b^Secondary;

Table 11.

*Model Coefficients for the indirect associations between Sociodemographic Factors on Interest in Internet delivery modality (imputed data).*

|  | Mediator | | | | | | | | | |
| --- | --- | --- | --- | --- | --- | --- | --- | --- | --- | --- |
| Antecedent | Self-efficacy | | Finding good health information | | Understanding health information | | Social Support | | Total | |
|  | B | [95% CI] | B | [95% CI] | B | [95% CI] | B | [95% CI] | B | [95% CI] |
| Age | -0.002 | [-0.001, 0.01] | -0.001 | [-0.01, 0.003] | <.001 | [-0.003, 0.004] | 0.003 | [-0.01, 0.02] | 0.001 | [-0.01, 0.01] |
| Gender | -0.01 | [-0.27, 0.07] | -0.02 | [-0.18, 0.12] | 0.0003 | [-0.09, 0.10] | 0.02 | [-0.11, 0.14] | 0.04 | [-0.25, 0.07] |
| Middle SES^a^ | 0.06 | [-0.04, 0.24] | -0.04 | [-0.19, 0.08] | 0.01 | [-0.10, 0.11] | -0.01 | [-0.09, 0.06] | - | - |
| High SES^a^ | 0.02 | [-0.09, 0.16] | -0.03 | [-0.18, 0.08] | 0.01 | [-0.10, 0.13] | -0.01 | [-0.10, 0.06] | - | - |
| BMI | -0.003 | [-0.01, 0.01] | .-0.001 | [-0.01, 0.01] | <.001 | [-0.01, 0.01] | 0.001 | [-0.01, 0.01] | -0.003 | [-0.02, 0.01] |
| TAFE^b^ | 0.02 | [-0.07, 0.15] | -0.08 | [-0.35, 0.10] | 0.02 | [-0.13, 0.17] | -0.01 | [-0.11, 0.08] | - | - |
| Tertiary^b^ | -0.02 | [-0.07, 0.17] | -0.03 | [-0.29, 0.08] | -0.01 | [-0.17, 0.24] | -0.02 | [-0.11, 0.08] | - | - |

Note: N=148, B is the unstandardized coefficient.

Reference levels: ^a^Low SES; ^b^Secondary.

-Indirect effects are not calculated for multinomial variables in PROCESS.
